# Duration of osteoporosis treatment to reduce the risk of subsequent osteoporotic fracture and all-cause mortality in elderly hip fracture patients in a Korean real-world study

**DOI:** 10.1007/s11657-024-01366-7

**Published:** 2024-01-10

**Authors:** Soong Joon Lee, Minjoon Cho, Hojoon Lee, Hyuna Lim, Jae Hyup Lee

**Affiliations:** 1 Department of Orthopedic Surgery, Seoul Metropolitan Government-Seoul National University Boramae Medical Center, Seoul, South Korea; 2 Department of Orthopedic Surgery, Seoul National University College of Medicine, Seoul, South Korea; 3 Amgen Korea Ltd., Seoul, South Korea

**Keywords:** All-cause mortality, Hip fracture, Osteoporosis, South Korea, Subsequent osteoporotic fracture, Treatment duration

## Abstract

**Summary:**

This study aimed to evaluate the association between treatment duration of osteoporosis medications and clinical outcomes of patients with hip fracture. We found that the risk of subsequent osteoporotic fractures and all-cause mortality showed a decreasing trend as the treatment duration of osteoporosis medications increased.

**Purpose:**

To assess the risk of subsequent osteoporotic fracture (SOF) and all-cause mortality (ACM) in elderly patients with hip fracture in South Korea and to evaluate the potential reduction in the risk of SOF and ACM with varying durations of osteoporosis treatment.

**Methods:**

Newly diagnosed patients with hip fracture (age ≥ 60 years) who initiated osteoporosis medication within 3 months after the hip fracture from 2003–2014 were identified from the National Health Insurance Service-Senior cohort. The risk of SOF and ACM was estimated after the 1-year exposure-measurement period. Adjusted hazard ratios (aHRs) were calculated for treatment duration of osteoporosis medications categorized as short-term treatment (ST, < 3 months), early discontinuation (ED, ≥ 3– < 6 months), late discontinuation (LD, ≥ 6– < 12 months), and treatment continuation (TC, ≥ 12 months).

**Results:**

A total of 4,421 patients were included in the analysis. The 3-year cumulative incidence of SOF was 22.4%, 22.0%, 23.9%, and 21.6%, and that of 3-year ACM was 29.8%, 27.0%, 19.7%, and 18.9% in the ST, ED, LD, and TC groups, respectively. Compared with the ST group, the risk of SOF showed a decreasing trend in the TC group (aHR [95% CI], 0.77 [0.58–1.00]). The risk of ACM was significantly reduced in the LD (aHR 0.68 [0.57–0.82]) and TC (aHR 0.65 [0.50–0.84]) groups.

**Conclusion:**

These findings underscore the importance of early and continuous osteoporosis treatment for elderly patients with hip fracture to improve health outcomes. The benefits of long-term osteoporosis treatment should be discussed in clinical practice to improve overall health outcomes.

**Supplementary Information:**

The online version contains supplementary material available at 10.1007/s11657-024-01366-7.

## Introduction

Hip fracture rates have attained epidemic proportions worldwide, particularly in the elderly population, and are a major public health concern [1]. Globally, the incidence of hip fracture was 14.2 million in 2019, an increase of approximately 93.0% since 1990 [1]. Among 271,197 South Korean women at the age of 66 years between January 1, 2008 and December 31, 2015 who were screened for bone mineral density, the 10-year cumulative incidence of fragility fractures was higher in women with osteoporosis (hazard ratio [HR] [95% confidence interval; CI]: 1.68 [1.64–1.72]) compared with women who had a normal bone mineral density [2]. With an increase in the aging population, the economic burden of hip fracture is predicted to increase further in the future [3]. Since the risk of hip fracture increases immediately after a preceding fracture [4], a multistakeholder coalition was assembled by the American Society for Bone and Mineral Research to develop clinical recommendations for the optimal prevention of secondary fractures among people aged ≥ 65 years with a hip or vertebral fracture [5]. The coalition recommended that pharmacological therapy for osteoporosis must be offered to individuals aged ≥ 65 years to reduce the risk of additional fractures [5].

In real-world studies conducted in patients with hip fracture, osteoporosis medications have reduced the incidence of fracture and all-cause mortality over time [6, 7]. Despite good evidence showing an increased risk of hip fracture in patients with osteoporosis, the mean rate of global treatment initiation with osteoporosis medications in patients with hip fracture is low (6.5%–33.5%) [8–12]. In Korea, approximately 42.0% of patients had initiated osteoporosis medication in the first 12 months after an osteoporotic fracture. Moreover, the rate of prescription of osteoporosis medications was lower in patients with a hip fracture than in those with a vertebral fracture (36.6% versus 53.2%) [13]. In a study conducted in the United States of America, 19.0%–26.0% of patients discontinued osteoporosis treatment at an early stage [14], potentially leading to a higher risk of subsequent fracture or death. Long-term osteoporosis treatment (> 1 year) might reduce the risk of fracture and all-cause mortality [6, 7]. In a real-world study from Spain, 28.3% of patients were prescribed an osteoporotic medication within 6 months since the hip fracture and 54.5% of naïve patients had discontinued the treatment 1 year after the fracture [15]. Since only a few studies have evaluated the impact of osteoporosis medication duration on the incidence of subsequent fracture or death after a hip fracture [16, 17], evidence on treatment duration focusing on early discontinuation and long-term continuation needs to be investigated.

Therefore, we proposed a study to evaluate the association between treatment duration of osteoporosis medications and clinical outcomes of patients with a hip fracture. We also questioned whether there was a difference in the risk of subsequent osteoporotic fracture (SOF) and mortality among patients who discontinued the osteoporosis treatment in the first year of treatment or who continued the treatment for more than 1 year according to the osteoporosis treatment duration. Consequently, this study aimed to estimate the risk of SOF and all-cause mortality following an initial hip fracture and to evaluate the risk reduction in SOF and all-cause mortality by osteoporosis treatment duration among the elderly patients with hip fracture in South Korea.

## Methods

### Study design

This retrospective cohort study used the National Health Insurance Service (NHIS)-Senior cohort, which consisted of 558,147 individuals selected using a 10.0% simple random sampling method from a total of 5.5 million individuals aged ≥ 60 years in 2002 from the National Health Information Database [18]. The data collection period of the Senior cohort was from January 1, 2002, to December 31, 2015. This cohort provided data on demographics, socioeconomic information, and utilization of medical services, including inpatient/ outpatient status, medical procedures, and prescriptions [18].

### Study population

Patients aged ≥ 60 years who were newly diagnosed with a hip fracture (using the International Classification of Diseases [ICD] 8th revision codes S72.0 and S72.1) and underwent hip fracture–related surgery between 2003 and 2014 were identified in the cohort. The procedure codes for hip fracture–related surgery are provided in Appendix 1 (Online Resource 1). We considered combining diagnosis and procedure codes as it is known to have a high validity in detecting a hip fracture [19]. Patients with a pathological fracture (ICD code: M90.7) prior to hip fracture–related surgery and those who had a hip fracture prior to December 31, 2002, were excluded.

### Exposure assessment

**Fig. 1:**
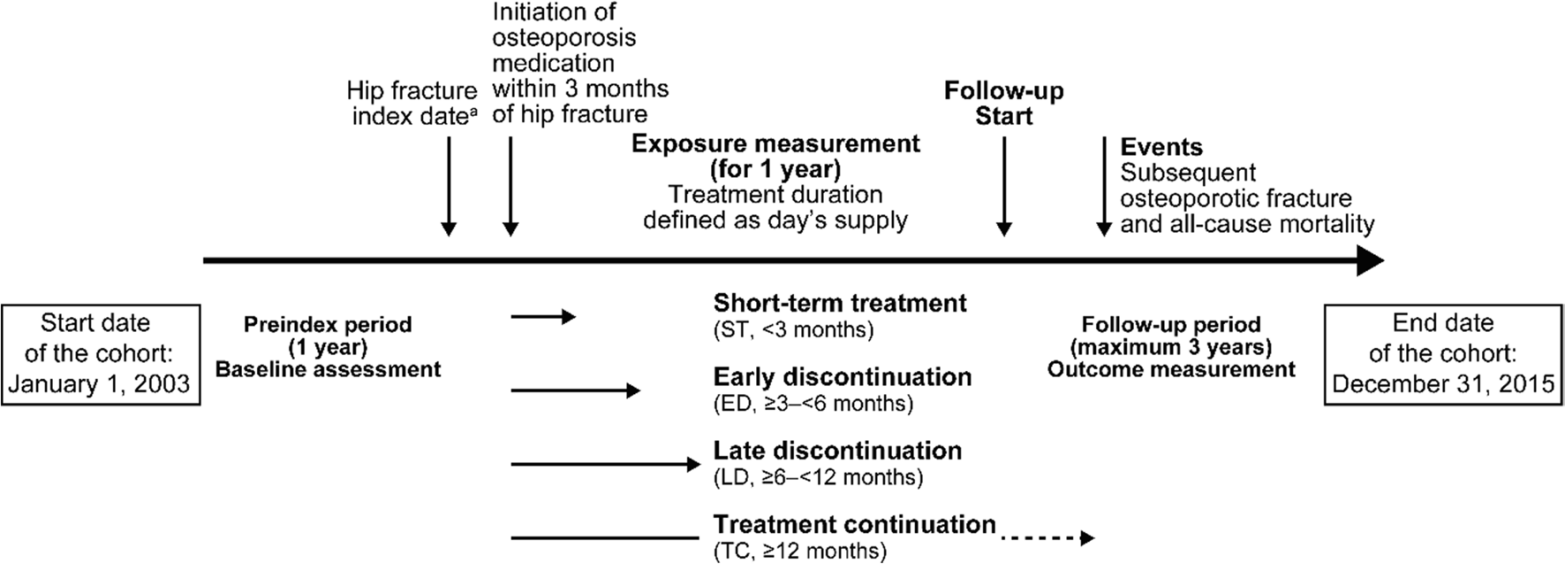
Study design schema. ^a^Data source: NHIS-Senior cohort patients aged ≥ 60 years in 2002 (10% of the Korean population within the age group; n = 558,147)

To ensure homogeneous baseline characteristics of patients with hip fracture, only patients who initiated osteoporosis medications within 3 months of a hip fracture diagnosis were included. Osteoporosis medications included bisphosphonates, bisphosphonates in combination with vitamin D, and selective estrogen receptor modulators, which were approved and reimbursed during the study period (Appendix 2, Online Resource 1). The index date was defined as the first prescription date of any osteoporosis medication within 3 months of the hip fracture date (Fig. 1).

The exposure of interest was osteoporosis treatment for 1 year from the index date. The continuous use of osteoporosis medications for 1 year after the first prescription date was assessed and the duration was categorized into four groups: short-term treatment (ST, < 3 months), early discontinuation (ED, ≥ 3– < 6 months), late discontinuation (LD, ≥ 6– < 12 months), and treatment continuation (TC, ≥ 12 months). The treatment duration was calculated based on the total number of supply days for 1 year regardless of drug switching. In the analysis, the permissible refill gap was considered to be 60 days.

### Covariates

Demographic information, including age, sex, residence, economic status, and disability, were collected in the index year. Comorbidities included diabetes mellitus (both type 1 and type 2), hypertension, dyslipidemia, coronary heart disease, cardiac arrhythmia, peripheral arterial occlusive disease, chronic kidney disease, Parkinson’s disease, chronic obstructive pulmonary disease, rheumatoid arthritis, cancer, liver dysfunction, stroke or transient ischemic attack, and dementia. Comorbidities were assessed within the baseline period, which began 12 months before the index date, and the Charlson comorbidity index (CCI; categories: 0, 1, 2, and ≥ 3) was also used to characterize the patient’s clinical history [20]. However, immune deficiency syndrome or human immunodeficiency virus were not included in the calculation of CCI as these are treated as sensitive information in accordance with the policy from the data provider (Appendix 3, Online Resource 1). Concomitant use of medications was considered when a patient had a history of prescription of drugs for other conditions during the treatment duration of osteoporosis medications.

### Outcomes

The primary outcomes were defined as SOF and all-cause mortality. We used 3-year cumulative incidence to describe the risk of these events. The SOFs were identified from the fracture diagnosis in either inpatient or outpatient claims databases using the ICD codes and included fractures of the hip, vertebra, proximal humerus, radius, ankle, pelvis, rib, distal femur, and clavicle (Appendix 4, Online Resource 1). To differentiate the new incident hip fracture, medical history coded for hip fracture within 6 months from the first diagnosis of a hip fracture was regarded as a follow-up care for the initial hip fracture. The SOF except for hip fracture was defined as a qualified fracture when there was a hospitalization event for more than 2 days or outpatient care at least 3 times with a predefined diagnosis code for 6 months. For each qualified fracture, the first date of claims was considered as the event date. Death events were defined as patients with an effective date of death in the dataset.

This study employed landmark analysis to consider immortal time bias and landmark time was selected at 1 year from the index date corresponding to the exposure measurement period. Patients who experienced the outcomes of interest during landmark 1-year exposure measurement period were excluded from the analysis. The follow-up of all study outcomes ended either at the date of occurrence of the outcome (SOF or all-cause mortality) or at the last follow-up date of the cohort (December 31, 2015), whichever was earlier.

### Statistical analysis

Patient characteristics with respect to demographics, clinical history, and concomitant medication use were summarized as mean (standard deviation [SD]) or median (interquartile range [IQR]) for continuous variables and as frequency and percentage for categorical variables. To estimate the reduction in the risk of SOF and all-cause mortality according to treatment duration, the early treatment period (ST group) was used as a negative control period (reference) and the remaining groups (ED, LD, and TC) were compared with the ST group. The incidence of SOF and all-cause mortality was estimated through stratification by the duration of osteoporosis medication use after the initial hip fracture, and the annual cumulative incidence for 1, 2, and 3 years was calculated using the Kaplan–Meier method. Kaplan–Meier survival curves were generated separately for SOF and all-cause mortality. A Cox proportional hazards model was used to estimate the effect of different treatment durations. Crude and adjusted HRs (aHRs) with 95% CIs were calculated for each exposure group. HRs were adjusted for index year, sex, age group, residence, income, type of insurance, disability, any osteoporotic fracture before the initial hip fracture, use of osteoporosis medications before the initial hip fracture, comorbidities, and concurrent medications listed in the Table 1.

**Table 1:** Demographics and baseline clinical characteristics of patients who initiated osteoporosis medication within 3 months from the date of the hip fracture

	ST group (n = 1,673)	ED group (n = 1,131)	LD group (n = 1,130)	TC group (n = 487)	*p* value
Time to treatment initiation (days)					
Mean ± SD	14.1 ± 22.6	16.6 ± 24.5	20.0 ± 25.1	23.0 ± 27.1	
Median (IQR)	0 (0–26)	0 (0–31)	0 (0–39)	7 (0–44)	< 0.0001
Demographics					
Patients, index year					< 0.0001
2003	63 (47.7)	30 (22.7)	34 (25.8)	5 (3.8)	
2004	101 (53.2)	46 (24.2)	30 (15.8)	13 (6.8)	
2005	112 (44.8)	52 (20.8)	61 (24.4)	25 (10.0)	
2006	158 (43.5)	93 (25.6)	83 (22.9)	29 (8.0)	
2007	150 (40.3)	90 (24.2)	117 (31.5)	15 (4.0)	
2008	171 (45.0)	83 (21.8)	85 (22.4)	41 (10.8)	
2009	177 (41.0)	112 (25.9)	119 (27.6)	24 (5.6)	
2010	143 (31.7)	120 (26.6)	117 (25.9)	71 (15.7)	
2011	147 (33.1)	126 (28.4)	99 (22.3)	72 (16.2)	
2012	167 (33.9)	125 (25.4)	121 (24.5)	80 (16.2)	
2013	150 (31.1)	137 (28.4)	138 (28.6)	58 (12.0)	
2014	134 (31.1)	117 (27.2)	126 (29.2)	54 (12.5)	
Sex					< 0.0001
Male	322 (49.3)	151 (23.1)	140 (21.4)	40 (6.1)	
Female	1,351 (35.9)	980 (26.0)	990 (26.3)	447 (11.9)	
Age, years					
Mean ± SD	79.6 ± 6.7	79.6 ± 6.4	78.0 ± 6.4	78.1 ± 6.7	
Median (IQR)	79 (75–84)	80 (75–84)	78 (73–83)	78 (73–83)	< 0.0001
60–64	12 (44.4)	6 (22.2)	7 (25.9)	2 (7.4)	< 0.0001
65–69	102 (34.1)	57 (19.1)	96 (32.1)	44 (14.7)	
70–74	281 (33.6)	190 (22.7)	244 (29.2)	122 (14.6)	
75–79	450 (37.2)	300 (24.8)	340 (28.1)	119 (9.8)	
80–84	438 (39.2)	322 (28.8)	249 (22.3)	108 (9.7)	
85–89	261 (38.8)	195 (29.0)	143 (21.3)	73 (10.9)	
90 +	129 (49.6)	61 (23.5)	51 (19.6)	19 (7.3)	
Residence					< 0.0001
Metropolis	655 (33.0)	522 (26.3)	537 (27.1)	270 (13.6)	
City	328 (41.5)	194 (24.5)	187 (23.6)	82 (10.4)	
Rural	690 (41.9)	415 (25.2)	406 (24.7)	135 (8.2)	
Income					0.003
Medical aid (the lowest level)	198 (36.3)	139 (25.5)	151 (27.7)	58 (10.6)	
1–4	432 (40.6)	286 (26.9)	242 (22.8)	103 (9.7)	
5–8	475 (39.4)	313 (26.0)	304 (25.2)	113 (9.4)	
9–10 (the highest level)	568 (35.4)	393 (24.5)	433 (26.9)	213 (13.3)	
Type of insurance					0.410
Medical aid	198 (36.3)	139 (25.5)	151 (27.7)	58 (10.6)	
Employee-insured	1,013 (38.4)	663 (25.1)	656 (24.8)	309 (11.7)	
Self-employed, insured	462 (37.4)	329 (26.7)	323 (26.2)	120 (9.7)	
Disability					0.065
None	1,622 (37.6)	1,101 (25.5)	1,116 (25.9)	478 (11.1)	
Mild disability (grades 3–6)	22 (51.2)	10 (23.3)	7 (16.3)	4 (9.3)	
Serious disability (grades 1–2)	29 (47.5)	20 (32.8)	7 (11.5)	5 (8.2)	
Any osteoporotic fracture before the first hip fracture (except for hip fracture)	509 (36.1)	359 (25.5)	370 (26.2)	172 (12.2)	0.1968
Osteoporosis medication use before the first hip fracture					
Ever used	620 (30.7)	507 (25.1)	619 (30.6)	274 (13.6)	< 0.0001
Used within 1 year	292 (26.7)	233 (21.3)	400 (36.6)	169 (15.5)	< 0.0001
Comorbidities					
Diabetes mellitus	427 (35.6)	314 (26.2)	331 (27.6)	128 (10.7)	0.154
Hypertension	990 (36.8)	672 (24.9)	730 (27.1)	302 (11.2)	0.020
Dyslipidemia	176 (30.0)	145 (24.7)	170 (29.0)	95 (16.2)	< 0.0001
Coronary heart disease	178 (34.9)	132 (25.9)	138 (27.1)	62 (12.2)	0.471
Cardiac arrhythmia	77 (33.6)	58 (25.3)	58 (25.3)	36 (15.7)	0.112
PAOD	149 (40.9)	90 (24.7)	77 (21.2)	48 (13.2)	0.120
Chronic kidney disease	27 (32.9)	20 (24.4)	28 (34.2)	7 (8.5)	0.327
Parkinson’s disease	50 (38.5)	38 (29.2)	27 (20.7)	15 (11.5)	0.584
COPD	223 (39.0)	148 (25.9)	152 (26.6)	49 (8.6)	0.252
Rheumatoid arthritis	70 (31.0)	50 (22.1)	70 (31.0)	36 (15.9)	0.007
Cancer	162 (38.2)	101 (23.8)	126 (29.7)	35 (8.3)	0.072
Liver dysfunction	97 (31.1)	82 (26.3)	99 (31.7)	34 (10.9)	0.028
Stroke or TIA	347 (37.0)	220 (23.5)	266 (28.4)	104 (11.1)	0.112
Dementia	264 (41.2)	183 (28.6)	138 (21.5)	56 (8.7)	0.004
CCI					0.055
0	441 (40.3)	293 (26.8)	252 (23.0)	109 (10.0)	
1	452 (37.2)	296 (24.4)	314 (25.8)	153 (12.6)	
2	327 (38.4)	213 (25.0)	211 (24.8)	101 (11.9)	
≥ 3	453 (36.0)	329 (26.1)	353 (28.0)	124 (9.9)	
Concomitant medication					
Acetaminophen	328 (23.4)	343 (24.4)	486 (34.6)	247 (17.6)	< 0.0001
Antidepressants	232 (27.8)	227 (27.2)	257 (30.8)	119 (14.3)	< 0.0001
Antiepileptics	86 (21.7)	94 (23.7)	149 (37.6)	67 (16.9)	< 0.0001
Antipsychotics	396 (29.0)	359 (26.2)	422 (30.9)	191 (14.0)	< 0.0001
Anxiolytics	461 (27.5)	418 (24.9)	554 (33.0)	245 (14.6)	< 0.0001
CNS stimulants	64 (20.4)	68 (21.7)	134 (42.7)	48 (15.3)	< 0.0001
COX-2 inhibitors	294 (24.4)	316 (26.2)	390 (32.3)	207 (17.2)	< 0.0001
HRT (women only)	3 (15.8)	6 (31.6)	8 (42.1)	2 (10.5)	0.190*
Hypnotics	192 (31.6)	159 (26.2)	170 (28.0)	87 (14.3)	0.001
Loop diuretics	152 (32.8)	126 (27.2)	124 (26.8)	61 (13.2)	0.091
Methotrexate	8 (27.6)	3 (10.3)	10 (34.5)	8 (27.6)	0.009
NSAIDs	1,406 (36.5)	973 (25.3)	1,036 (26.9)	439 (11.4)	< 0.0001
PPIs	124 (21.9)	126 (22.3)	212 (37.5)	104 (18.4)	< 0.0001
Steroids (including corticosteroids)	119 (15.6)	183 (24.0)	312 (40.8)	150 (19.6)	< 0.0001
Thiazolidinediones	14 (29.2)	16 (33.3)	15 (31.3)	3 (6.3)	0.293
Thyroid therapy^a^	22 (29.3)	14 (18.7)	25 (33.3)	14 (18.7)	0.033

Percentages are calculated using the total number of patients in the ST, ED, LD, and TC groups as the denominator

Data are presented as n (%) unless otherwise specified

ST group, < 3 months of treatment with osteoporosis medications; ED group, ≥ 3– < 6 months of treatment with osteoporosis medications; LD group, ≥ 6– < 12 months of treatment with osteoporosis medications; and TC group, ≥ 12 months of treatment with osteoporosis medications

*CCI*, Charlson Comorbidity Index; *CNS*, central nervous system; *COPD*, chronic obstructive pulmonary disease; *COX*, cyclooxygenase; *ED*, early discontinuation; *HRT*, hormone replacement therapy; *IQR*, interquartile range; *LD*, late discontinuation; *NSAID*, nonsteroidal anti-inflammatory drug; *PAOD*, peripheral arterial occlusive disease; *PPI*, proton pump inhibitor; *SD*, standard deviation; *ST*, short-term treatment; *TC*, treatment continuation; *TIA*, transient ischemic attack

^a^Includes levothyroxine sodium and a combination of levothyroxine and liothyronine sodium indicated for the treatment of hypothyroidism

^*^Fisher’s exact test

Subgroup analysis was conducted to determine the cumulative incidence of SOF and all-cause mortality by sex (male and female), site of SOF (vertebral, hip, and nonvertebral nonhip), and cause-specific death (diseases of the respiratory system; diseases of the circulatory system; and endocrine, nutritional, and metabolic diseases).

We performed several sensitivity analyses. First, multiple-treatment propensity scores weighted analysis using the Toolkit for Weighting and Analysis of Nonequivalent Groups (TWANG) was performed and based on the same covariates included in the covariate adjustment model [21]. Given the assumption that the propensity score should be considered based on variables affecting pre-treatment, covariates related to concurrent medication use were additionally examined within the baseline period, coinciding with the assessment period of comorbidities. The balance for any covariates was determined by standardized effect size < 0.10. Then, weighted HRs (wHRs) with 95% CIs were presented using the weighted Cox proportional hazards model. Second, since prior fractures may lead patients to be susceptible to a subsequent fracture, restricted analysis was carried out for event-free patients who had not experienced any SOF within 3 years prior to the first hip fracture. Last, in order to explore the residual effects and misclassification errors resulting from converting a continuous variable into a categorical variable, we fitted the treatment duration itself, measured as a continuous variable, to the Cox proportional hazard model.

All planned analyses were performed using RStudio version 3.3.3 and SAS Enterprise Guide version 7.1 in the remote system provided by the NHIS.

## Results

### Patient flow

**Fig. 2:**
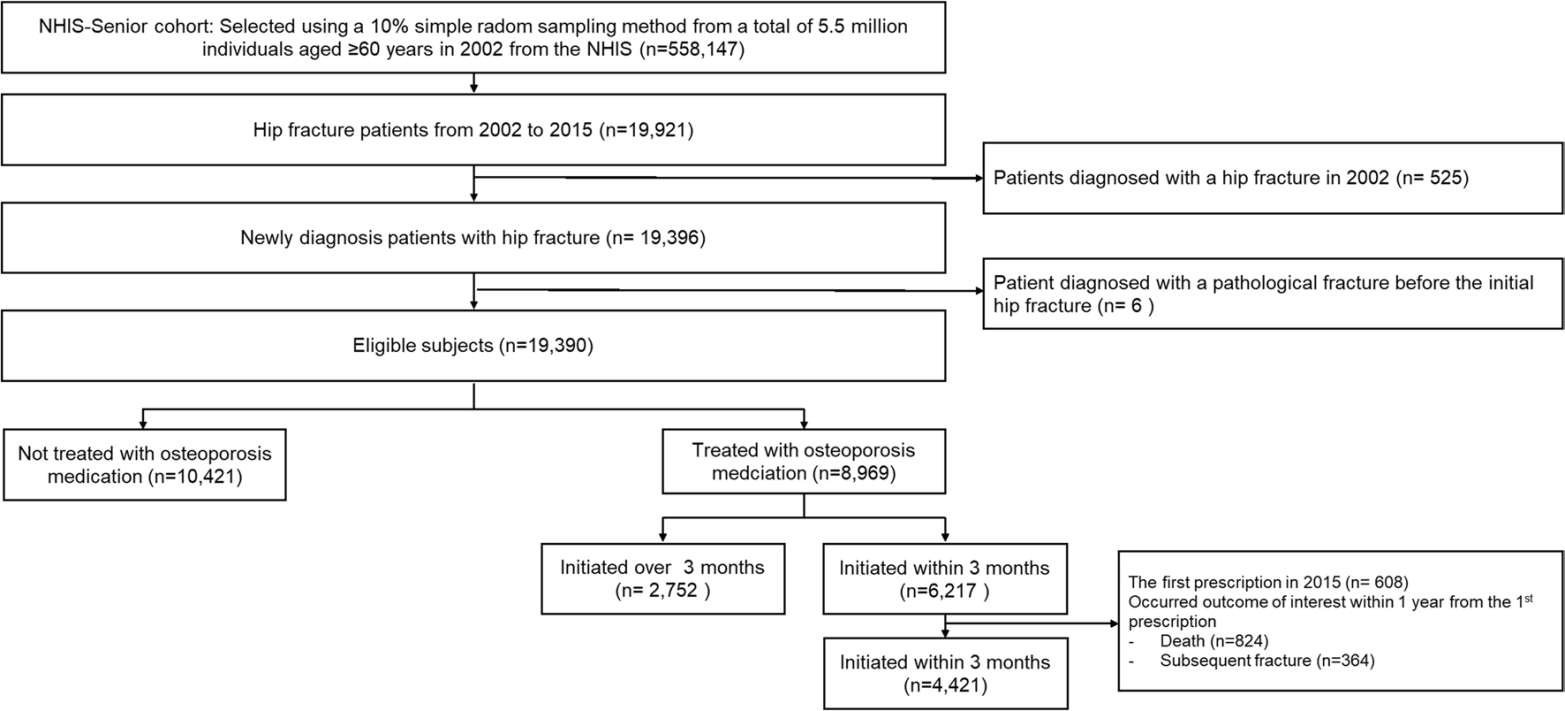
Patient flow diagram. *NHIS*, National Health Insurance Service

Of the 558,147 patients from the NHIS-Senior cohort, 19,390 patients were identified as newly diagnosed with hip fracture without pathological fracture during the study time. After excluding patients without osteoporosis treatment or those who initiated treatment after 3 months from the date of the hip fracture, 6,217 patients were identified as patients who initiated treatment within 3 months of the hip fracture. Patients who had received the first prescription for an osteoporosis medication in 2015 or those who experienced the outcome of interest (SOF or all-cause mortality) during the exposure-measurement period were excluded and 4,421 patients were included in the final analysis (Fig. 2). A comparison of demographics and baseline clinical characteristics between two groups by the treatment initiation timing was provided in Supplementary Table 1 (Online Resource 1).

### Demographics and baseline clinical characteristics

Of the patients who initiated osteoporosis medication within 3 months, approximately 85.0% were female and the mean age was 79.0 years. Among the 4,421 patients who initiated osteoporosis medication within 3 months, 1,673 (37.8%) were included in the ST group, 1,131 (25.6%) in the ED group, 1,130 (25.6%) in the LD group, and 487 (11.0%) in the TC group. The mean (SD) time to treatment initiation was 14.1 (22.6), 16.6 (24.5), 20.0 (25.1), and 23.0 (27.1) days in the ST, ED, LD, and TC groups, respectively (Table 1). Patients in the ST group were more likely to have comorbidities than those in the ED, LD, and TC groups. The prevalence of hypertension, dyslipidemia, rheumatoid arthritis, liver dysfunction, and dementia was significantly different among the four treatment groups (Table 1).

### Cumulative incidence of SOF and all-cause mortality

**Fig. 3:**
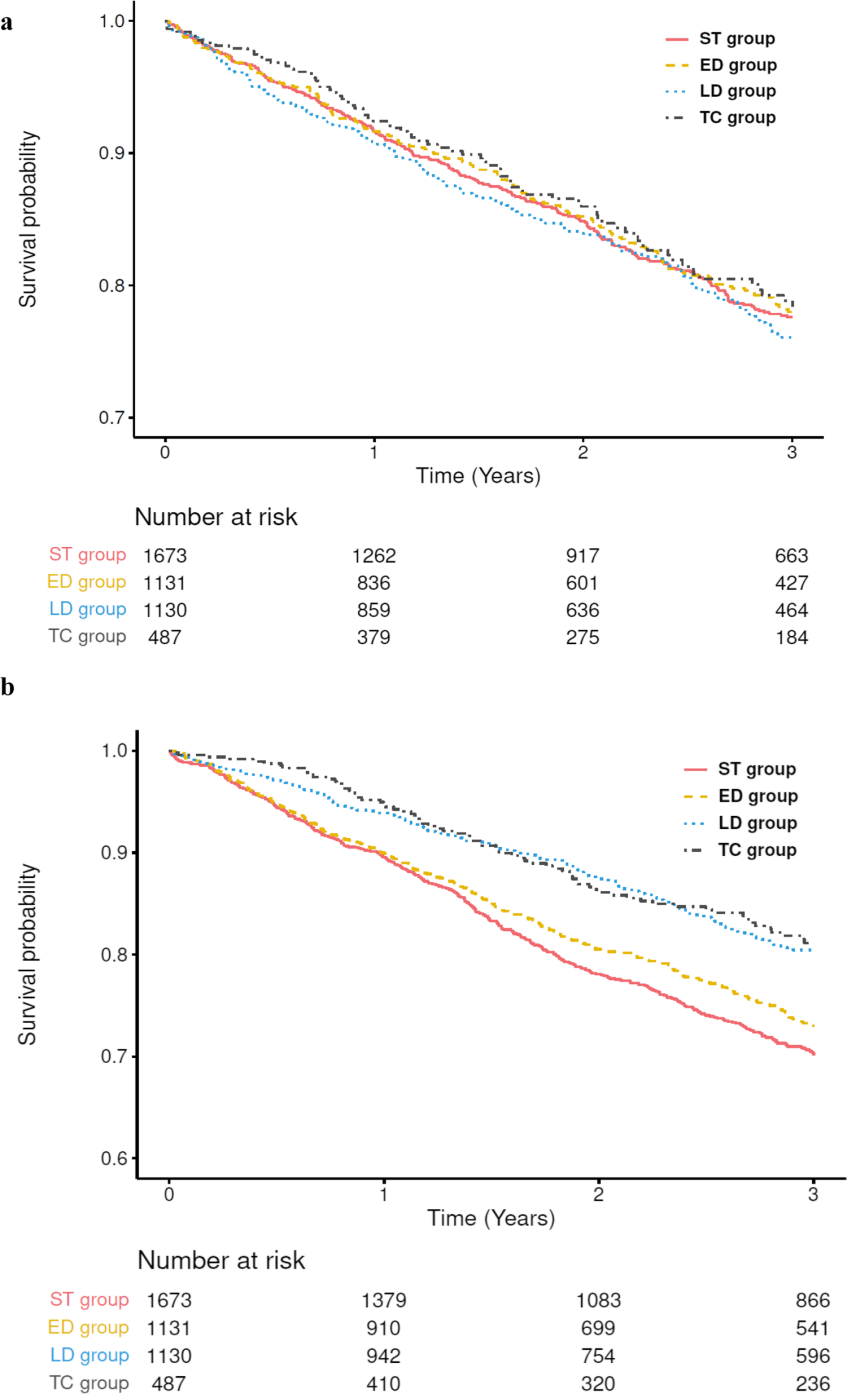
Kaplan–Meier curve for the incidence of (**a**) SOF and (**b**) all-cause mortality. ST group, < 3 months of treatment with osteoporosis medications; ED group, ≥ 3– < 6 months of treatment with osteoporosis medications; LD group, ≥ 6– < 12 months of treatment with osteoporosis medications; and TC group, ≥ 12 months of treatment with osteoporosis medications. *ED*, early discontinuation; *LD*, late discontinuation; *SOF*, subsequent osteoporotic fracture; *ST*, short-term treatment; *TC*, treatment continuation

The cumulative incidence (95% CI) of SOF at 3 years by the treatment duration of osteoporosis medications was 22.4% (20.1–24.9) in the ST group, 22.0% (19.3–25.1) in the ED group, 23.9% (21.2–27.0) in the LD group, and 21.6% (17.6–26.4) in the TC group (Fig. 3a). The 3-year cumulative incidence of SOF was higher in females than in males. The cumulative incidence (95% CI) of SOF at 3 years in females versus males was 23.4 (20.9–26.2) versus 17.5 (12.9–23.3) in the ST group, 22.6 (19.6–25.9) versus 18.3 (11.9–27.6) in the ED group, 24.9 (22.0–28.2) versus 15.4 (9.5–24.4) in the LD group, and 22.0 (17.8–27.0) versus 14.6 (6.3–31.7) in the TC group.

The cumulative incidence (95% CI) of all-cause mortality at 3 years was 29.8% (27.6–32.3) in the ST group, 27.0% (24.2–30.0) in the ED group, 19.7% (17.2–22.4) in the LD group, and 18.9% (15.3–23.1) in the TC group (Fig. 3b). The 3-year cumulative incidence of all-cause mortality was higher in males than in females. The cumulative incidence (95% CI) of all-cause mortality at 3 years in males versus females was 31.5% (26.3–37.4) versus 29.4% (26.9–32.1) in the ST group, 37.4% (29.8–46.2) versus 25.3% (22.4–28.5) in the ED group, 37.5% (29.2–47.3) versus 17.2% (14.8–20.0) in the LD group, and 33.5% (20.5–51.7) versus 17.4% (13.8–21.8) in the TC group.

### Effect of the treatment duration on SOF and all-cause mortality

Compared with patients in the ST group, those in the TC group had a trend of reduced risk of SOF (aHR [95% CI], 0.77 [0.58–1.00]). The risk of SOF was significantly reduced by 35.0% for vertebral fractures (aHR [95% CI], 0.65 [0.44–0.96]), by 21.0% for hip fractures (aHR [95% CI], 0.79 [0.44–1.42]), and by 1.0% for nonvertebral nonhip fractures (aHR [95% CI], 0.99 [0.67–1.47]) in the TC group compared with that in the ST group (Table 2). The risk of subsequent vertebral fracture was also significantly reduced in females (aHR [95% CI], 0.61 [0.41–0.91]) but not males (aHR [95% CI], 1.21 [0.18–7.95]).

**Table 2:** Effect of treatment duration with osteoporosis medications on risk of subsequent osteoporotic fracture

	SOF		Vertebral		Hip		Nonvertebral/nonhip	
Treatment duration	Crude HR	Adjusted HR*	Crude HR	Adjusted HR*	Crude HR	Adjusted HR*	Crude HR	Adjusted HR*
All								
ST group	Reference	Reference	Reference	Reference	Reference	Reference	Reference	Reference
ED group	0.98 (0.81–1.17)	0.94 (0.77–1.13)	1.03 (0.79–1.34)	0.96 (0.73–1.25)	0.94 (0.64–1.39)	0.93 (0.62–1.39)	1.07 (0.81–1.42)	1.04 (0.78–1.39)
LD group	1.08 (0.90–1.29)	0.94 (0.77–1.14)	1.05 (0.81–1.36)	0.82 (0.62–1.09)	1.10 (0.76–1.59)	1.03 (0.69–1.55)	1.26 (0.96–1.65)	1.11 (0.83–1.49)
TC group	0.93 (0.73–1.20)	0.77 (0.58–1.00)	0.95 (0.66–1.35)	0.65 (0.44–0.96)	0.82 (0.47–1.42)	0.79 (0.44–1.42)	1.15 (0.80–1.65)	0.99 (0.67–1.47)
Male								
ST group	Reference	Reference	Reference	Reference	Reference	Reference	Reference	Reference
ED group	0.97 (0.56–1.67)	1.24 (0.67–2.28)	1.50 (0.67–3.39)	2.27 (0.84–6.15)	0.87 (0.36–2.13)	1.13 (0.39–3.34)	1.01 (0.38–2.68)	1.30 (0.42–4.10)
LD group	0.90 (0.51–1.62)	1.21 (0.60–2.43)	1.13 (0.46–2.80)	1.35 (0.42–4.38)	0.85 (0.33–2.17)	0.98 (0.28–3.41)	1.54 (0.63–3.76)	2.97 (0.94–9.39)
TC group	1.03 (0.41–2.61)	1.14 (0.39–3.35)	1.12 (0.26–4.94)	1.21 (0.18–7.95)	0.52 (0.07–3.93)	0.96 (0.10–9.00)	1.45 (0.33–6.50)	1.42 (0.22–9.24)
Female								
ST group	Reference	Reference	Reference	Reference	Reference	Reference	Reference	Reference
ED group	0.96 (0.79–1.17)	0.94 (0.77–1.15)	0.96 (0.73–1.27)	0.91 (0.68–1.22)	0.98 (0.63–1.51)	0.96 (0.61–1.51)	1.05 (0.78–1.42)	1.05 (0.77–1.42)
LD group	1.07 (0.89–1.30)	0.94 (0.77–1.16)	1.00 (0.76–1.32)	0.79 (0.59–1.07)	1.19 (0.80–1.79)	1.05 (0.67–1.64)	1.20 (0.91–1.59)	1.11 (0.82–1.51)
TC group	0.90 (0.69–1.17)	0.74 (0.56–0.98)	0.88 (0.61–1.28)	0.61 (0.41–0.91)	0.90 (0.51–1.61)	0.81 (0.43–1.50)	1.07 (0.74–1.56)	0.96 (0.64–1.45)

Data are presented as HR with 95% confidence interval

ST group, < 3 months of treatment with osteoporosis medications; ED group, ≥ 3– < 6 months of treatment with osteoporosis medications; LD group, ≥ 6– < 12 months of treatment with osteoporosis medications; and TC group, ≥ 12 months of treatment with osteoporosis medications

*ED*, early discontinuation; *HR*, hazard ratio; *LD*, late discontinuation; SOF, subsequent osteoporotic fracture; *ST*, short-term treatment; *TC*, treatment continuation

^*^Adjusted for the index year, sex, age group, residence, income, type of insurance, disability, any osteoporotic fracture before the initial hip fracture, use of osteoporosis medications before the initial hip fracture, comorbidities, and concurrent medications listed in the baseline table

Patients in the TC group had a significantly reduced risk of all-cause mortality compared with those in the ST group (aHR [95% CI], 0.65 [0.50–0.84]). Patients in the LD group also showed a reduced risk of all-cause mortality versus those in the ST group (aHR [95% CI], 0.68 [0.57–0.82]). Male patients in the TC or LD group did not have a significantly reduced risk of all-cause mortality compared with those in the ST group. In contrast, the risk of all-cause mortality was significantly reduced in females in the TC (aHR [95% CI], 0.59 [0.44–0.78]) and LD (0.62 [0.50–0.77]) groups. The risk of death due to diseases of the circulatory system was significantly lower in patients in the TC group (aHR [95% CI], 0.34 [0.18–0.63]) and LD group (0.61 [0.43–0.88]) versus those in the ST group (Table 3). When stratified by sex, the reduction in the risk of death was significant only for females (TC group: aHR [95% CI], 0.30 [0.15–0.58] and LD group: aHR [95% CI], 0.59 [0.40–0.86]).

**Table 3:** Effect of treatment duration with osteoporosis medications on mortality

	All-cause mortality		Death due to diseases of the respiratory system		Death due to diseases of the circulatory system		Death due to endocrine, nutritional, and metabolic diseases	
Treatment duration	Crude HR	Adjusted HR*	Crude HR	Adjusted HR*	Crude HR	Adjusted HR*	Crude HR	Adjusted HR*
All								
ST group	Reference	Reference	Reference	Reference	Reference	Reference	Reference	Reference
ED group	0.89 (0.76–1.04)	0.90 (0.77–1.06)	0.92 (0.58–1.46)	0.90 (0.56–1.46)	0.85 (0.64–1.13)	0.87 (0.64–1.17)	0.68 (0.35–1.31)	0.63 (0.31–1.28)
LD group	0.60 (0.51–0.72)	0.68 (0.57–0.82)	0.75 (0.47–1.22)	0.82 (0.48–1.39)	0.52 (0.37–0.73)	0.61 (0.43–0.88)	0.50 (0.24–1.03)	0.48 (0.22–1.03)
TC group	0.58 (0.45–0.74)	0.65 (0.50–0.84)	0.63 (0.31–1.29)	0.59 (0.27–1.28)	0.31 (0.17–0.56)	0.34 (0.18–0.63)	0.70 (0.29–1.68)	0.67 (0.25–1.80)
Male								
ST group	Reference	Reference	Reference	Reference	Reference	Reference	Reference	Reference
ED group	1.19 (0.84–1.67)	1.24 (0.84–1.82)	1.42 (0.61–3.33)	1.66 (0.51–5.38)	0.91 (0.40–2.09)	0.90 (0.32–2.52)	0.34 (0.04–2.85)	NA
LD group	1.10 (0.76–1.58)	1.01 (0.64–1.57)	1.37 (0.57–3.30)	1.34 (0.31–5.86)	0.87 (0.37–2.09)	0.81 (0.23–2.82)	1.14 (0.29–4.58)	NA
TC group	1.10 (0.60–2.01)	1.21 (0.60–2.44)	0.61 (0.08–4.70)	2.75 (0.21–35.5)	0.90 (0.21–3.88)	0.74 (0.10–5.47)	1.39 (0.17–11.58)	NA
Female								
ST group	Reference	Reference	Reference	Reference	Reference	Reference	Reference	Reference
ED group	0.85 (0.71–1.00)	0.84 (0.71–1.01)	0.83 (0.48–1.43)	0.80 (0.45–1.44)	0.83 (0.61–1.12)	0.84 (0.61–1.15)	0.75 (0.37–1.51)	0.68 (0.31–1.46)
LD group	0.54 (0.44–0.65)	0.62 (0.50–0.77)	0.65 (0.37–1.17)	0.73 (0.39–1.38)	0.48 (0.33–0.68)	0.59 (0.40–0.86)	0.41 (0.18–0.96)	0.39 (0.16–0.96)
TC group	0.54 (0.41–0.70)	0.59 (0.44–0.78)	0.69 (0.32–1.48)	0.54 (0.23–1.29)	0.27 (0.14–0.51)	0.30 (0.15–0.58)	0.65 (0.25–1.72)	0.55 (0.19–1.65)

Data are presented as HR with 95% confidence interval

ST group, < 3 months of treatment with osteoporosis medications; ED group, ≥ 3– < 6 months of treatment with osteoporosis medications; LD group, ≥ 6– < 12 months of treatment with osteoporosis medications; and TC group, ≥ 12 months of treatment with osteoporosis medications

*CI*, confidence interval; *ED*, early discontinuation; *HR*, hazard ratio; *LD*, late discontinuation; *NA*, not available; *ST*, short-term treatment; *TC*, treatment continuation

^*^Adjusted for the index year, sex, age group, residence, income, type of insurance, disability, any osteoporotic fracture before the initial hip fracture, use of osteoporosis medications before the initial hip fracture, comorbidities, and concurrent medications listed in the baseline table

### Sensitivity analysis

The multiple treatment propensity score-weighted analysis generated results that were similar to the main findings obtained with the covariate-adjusted model. These results indicated that patients in the TC group had a lower risk compared with those in the ST group for SOF and mortality (wHR [95% CI], 0.84 [0.75–0.93], 0.63 [0.57–0.70]). (Supplementary Table 2, Online Resource 1).

Among excluding patients who have suffered any osteoporotic fracture within 3 years prior to initial hip fracture, the risk of SOF and mortality on treatment duration had a decreased trend but there was no significant difference for SOF in the adjustment model (Supplementary Table 3 and 4, Online Resource 1).

In the analysis where treatment duration was considered as a continuous supply of days rather than grouped, the results remained consistent with the main findings. A year increase in osteoporosis treatment was associated with a reduced risk of SOF and death (aHR [95% CI], 0.80 (0.65–1.00), 0.61 (0.49–0.75), respectively) (Supplementary Table 5, Online Resource 1).

## Discussion

The findings from this study revealed that approximately 30% of patients (6,217/19,390) with a hip fracture who were treated with osteoporosis medications initiated the treatment within 3 months of the initial fracture. The risk of SOF showed a trend toward reduction in patients in the TC group, and the risk of all-cause mortality was significantly reduced in patients in the TC and LD groups compared with those in the ST group. A reduced risk of SOF and all-cause mortality was associated with longer treatment with osteoporosis medications from the treatment initiation. Consequently, there is a need to increase the implementation of long-term prescription of osteoporosis medications in South Korea for patients who are predisposed to hip fractures.

Patients with more than 12 months of treatment with osteoporosis medications (TC group) showed a decreased risk of SOF compared with those who received treatment for less than 3 months (ST group). Our results corroborate the findings of previous studies that reported a lower risk of recurrent fractures in patients with hip fracture who took osteoporosis medications long-term [16, 17, 22]. In the TC group, the risk of subsequent vertebral fracture was significantly reduced by 35% and that of hip fracture was reduced by 21%; the reduction in the risk of hip fracture was not statistically significant. Our findings were similar to those reported in trials of a few osteoporosis medications where a significant reduction was noted in the risk of subsequent vertebral fracture but not subsequent hip fracture [23, 24]. However, there are several types of osteoporosis medications and each of these medications has a different potency, which can affect the efficacy in the prevention of fractures [25]. For example, the use of certain osteoporosis medications decreased the risk of subsequent hip fracture in patients aged > 80 years [26]. In a meta-analysis that evaluated the efficacy of osteoporosis treatment in patients aged > 75 years, osteoporosis treatment was significantly associated with a decreased risk of hip fracture at 1 and 3 years [27]. Thus, to reduce the risk of subsequent hip fractures, it is essential to choose the appropriate type of osteoporosis medication.

In our study, the risk of all-cause mortality was significantly lower in the LD and TC groups compared with the ST group. Treatment with osteoporosis medications can reduce mortality in patients with hip fractures [28, 29]. The effect of treatment duration on cause-specific mortality stratified by the leading cause of death was statistically significant only for diseases of the circulatory system in the LD and TC groups. Osteoporosis and diseases of the circulatory system coexist in elderly patients, and there is evidence of a potential causal link between cardiovascular disease and bone loss [30]. However, additional analysis of the effect of bisphosphonates on mortality due to atherosclerotic cardiovascular disease revealed that the effect was not statistically significant. These findings concord with those of a meta-analysis that concluded that bisphosphonates do not have beneficial or harmful effects on atherosclerotic cardiovascular events [31]. As the cause of death in the elderly population is difficult to determine, it may be difficult to estimate the treatment effect of osteoporosis medications on cause-specific death. The reduced risk of mortality in observational studies such as this needs to be interpreted with caution as the results may be confounded by the high adherence rates to osteoporosis medications [32]. Nevertheless, this study supports the preventive effect of continuation of osteoporosis medications in reducing all-cause mortality.

The patients in the LD and TC groups who continued using osteoporosis medications for ≥ 6 months had a reduced risk of all-cause mortality than those in the ST group who were treated with osteoporosis medications for < 3 months. To the best of our knowledge, this is the first study to show a reduced risk of all-cause mortality but not SOF with a treatment duration of ≥ 6 months compared with a treatment duration of < 3 months. Patients’ adherence or compliance to osteoporosis medications is one of the most important factors to reduce the risk of fracture or mortality [16, 17, 33]. Previous studies showed that treatment duration of > 12 months with good adherence reduced the risk of SOF in patients with osteoporosis medications after hip fractures [16, 17]. Compared with the risk of SOF, a decrease in the risk of all-cause mortality was observed after the treatment period of ≥ 6 months, suggesting that a potential threshold for the treatment period that reduced the risk of all-cause mortality might be shorter than that for reducing the risk of SOF. The differences in the effects of osteoporosis medications may be attributed to the differences in insurance reimbursement policies, eligibility by country, time and insurance type. Further well-designed studies are warranted to evaluate the exact threshold for the duration of osteoporosis medications to reduce the risk of all-cause mortality. Nonetheless, it is important to continue treatment with osteoporosis medications to reduce the risk of SOF and all-cause mortality. In our study, treatment duration of > 6 months for osteoporosis medications did not significantly reduce the risk of SOF; however, it reduced the risk of all-cause mortality.

One of the strengths of this study is that we use of a nationwide representative population-based cohort. The incidence of both SOF and all-cause mortality was estimated in our study. A detailed cause of death was not provided due to the possibility of personal identification and because the number of patients associated with each cause of death was limited. Nevertheless, our study is meaningful in that the cause of death was analyzed. The study also applied a validated algorithm to identify outcome events to ensure the accuracy and completeness of claims-based information. Lastly, rigorous epidemiological methods have been used to reduce bias and confounding. By excluding patients who initiated the treatment after 3 months, we could minimize the confounding by different initiation times; by excluding the patients who experienced SOF or mortality within 1 year after the index date, we could minimize the immortal person-time. Moreover, we employed an additional propensity score-weighted model in a sensitivity analysis to minimize residual confounding in the regression analysis. Our study has a few limitations including the potential for unmeasured confounding due to a lack of clinical information, such as bone mineral density, body mass index, and incidence of fragility or traumatic fracture. Since the many clinical guidelines recommend initiating treatment regardless of BMD score in patients with an osteoporotic fragility fracture, we specifically included patients who began treatment within 3 months of a hip fracture [34]. Therefore, we believed that narrowed patient inclusion criteria can minimize the effect of potential for unmeasured confounding due to BMD. The sample size was insufficient to draw meaningful conclusions in some subgroups, especially in males. Factors related to treatment interruption, such as deterioration of existing health conditions and renal dysfunction, were not analyzed. However, as no association was found in the cause-specific mortality analysis, there is little possibility of discontinuation due to poor health.

## Conclusion

The initial treatment rate with osteoporosis medications was low among elderly patients with hip fracture in South Korea. Compared with patients whose treatment duration was < 3 months (ST group), patients with treatment duration ≥ 6 months showed reduced all-cause mortality and patients with treatment duration ≥ 12 months showed reduced all-cause mortality and SOF. Furthermore, these results were evident in females compared with males and for a second vertebral fracture compared with other types of fractures. A longer duration of treatment with osteoporosis medications was beneficial in reducing the risk of SOF and all-cause mortality in elderly patients with hip fracture in Korean routine clinical practice. These findings underscore the importance of early and continuous osteoporosis treatment for elderly patients with hip fracture to improve health outcomes. The benefits of long-term osteoporosis treatment should be discussed in clinical practice to reduce the burden of disease and improve overall health outcomes.

## Supplementary Information

Below is the link to the electronic supplementary material.

Supplementary file1 (DOCX 158 KB)

## Data Availability

Data used in this study are available from the National Health Insurance Service (https://nhiss.nhis.or.kr/bd/ab/bdaba008cv.do).
