# Supplementary material for: Duration of osteoporosis treatment to reduce the risk of subsequent osteoporotic fracture and all-cause mortality in elderly hip fracture patients in a Korean real-world study
Source: Arch Osteoporos. 2024 Jan 10;19(1):9. doi: 10.1007/s11657-024-01366-7 (PMC10781800; doi:10.1007/s11657-024-01366-7)

**Online resource**

**Article title:** Original Article

**Journal name:** Osteoporosis International

**Title:** Duration of osteoporosis treatment to reduce the risk of subsequent osteoporotic fracture and all-cause mortality in elderly hip fracture patients in a Korean real-world study

**Author information:**

Soong Joon Lee^1,2^, Min Joon Cho^1,2^, Hojoon Lee^3^, Hyuna Lim^3^, Jae Hyup Lee^1,2^

^1^Department of Orthopedic Surgery, Seoul Metropolitan Government-Seoul National University Boramae Medical Center, Seoul, South Korea

^2^Department of Orthopedic Surgery, Seoul National University College of Medicine, Seoul, South Korea

^3^Amgen Korea Ltd., Seoul, South Korea

**Corresponding author**

Jae Hyup Lee

**Address:** 20 Boramae-ro 5-gil, Dongjak-gu, Seoul, South Korea

**Email:** [spinelee@snu.ac.kr](mailto:spinelee@snu.ac.kr)

**Phone:** +82-2-870-2314

**Fax:** +82-2-870-2889

**ORCID:** 0000-0002-2141-0266

**Appendix 1** Korea-specific procedure codes for hip fracture–related surgery

| Code | Description |
| --- | --- |
| N0715/N2710 | Hemiarthroplasty – Hip |
| N0711/N2070 | Total Hip Arthroplasty |
| N0981 | External Fixation – Pelvis, Femur |
| N0601/N0611 | Open Reduction of Fractured Extremity (ORIF) – Femur |
| N0991 | Closed Pinning – Femur |
| N0641 | Closed Reduction of Fractured Extremity – Pelvis, Femur |
| N0654 | Bone Traction – Skin Traction |
| N0652 | Bone Traction – Skeletal Traction |

Appendix 2 Osteoporosis medications covered by NHIS from 2002 to 2015

| Category | ATC code | Description | Administration route |
| --- | --- | --- | --- |
| Bisphosphonate | M05BA03 | Pamidronic acid | Oral/intravenous |
|  | M05BA04 | Alendronic acid | oral |
|  | M05BA06 | Ibandronic acid | Oral/intravenous |
|  | M05BA07 | Risedronic acid | oral |
|  | M05BA08 | Zoledronic acid | Intravenous |
| Bisphosphonates, combinations | M05BB03 | Alendronic acid and colecalciferol | Oral |
|  | M05BB07 | Risedronic acid and colecalciferol | Oral |
|  | M05BB09 | Ibandronic acid and colecalciferol | Oral |
| Selective estrogen receptor modulators | G03XC01 | Raloxifene | Oral |
|  | G03XC02 | Bazedoxifene | Oral |

*ATC*, Anatomical, Therapeutic Chemical; *NHIS*, National Health Insurance Service

**Appendix 3** Comorbidities and International Classification of Diseases (ICD, 10th revision) diagnosis codes in patients with hip fracture in Korea

| Comorbidities | ICD code |
| --- | --- |
| Diabetes | E10, E11, E12, E13, E14 |
| Hypertension | I10, I11, I12, I13, I15 |
| Dyslipidemia | E78.0, E78.1, E78.2, E78.3, E78.4, E78.5 |
| Coronary heart disease | I20, I21, I22, I23, I24, I25 |
| Cardiac dysrhythmia | I44.1, I44.2, I44.3, I45.6, I45.9, I47, I48, I49, R00.0, R00.1, R00.8, T82.1, Z45.0, Z95.0 |
| Peripheral arterial occlusive disease | K55.1, I70, I73.1, I73.8, I73.9, I77.1, Z95.9, Z95.82 |
| Kidney dysfunction | I12.0, I13.1, N03.2, N03.3, N03.4, N03.5, N03.6, N03.7, N05.2, N05.3, N05.4, N05.5, N05.6, N05.7, N25.0, N18, N19, Z49.0, Z49.1, Z49.2, Z94.0, Z99.2 |
| Parkinson disease | G20, G31.83 |
| Chronic obstructive pulmonary disease | J41, J42, J43, J44, J98.2, J98.3 |
| Rheumatoid arthritis | M05, M06 |
| Cancer | C** |
| Liver dysfunction | K70, K71, K72, K73, K74, K75, K76, K77, B15, B16, B17, B18, B19 |
| Stroke or transient ischemic attack | G45, G46, I60, I61, I62, I63, I64, I65, I66, I67, I68, I69, H34.0 |
| Dementia | F00, F01, F02, F03, F05.1, G30, G31.1 |

*ICD*, International Classification of Diseases

**Appendix 4** International Classification of Diseases (ICD, 10th revision) codes to define the subsequent osteoporotic fracture

| Site | ICD code | Description |
| --- | --- | --- |
| Vertebral | M48.4 | Fatigue fracture of vertebra |
|  | M48.5 | Collapsed vertebra, NEC |
|  | S22.0 | Fracture of thoracic vertebra |
|  | S22.1 | Multiple fractures of thoracic spine |
|  | S32.0 | Fracture of lumbar vertebra |
|  | S32.1 | Fracture of sacrum |
|  | S32.2 | Fracture of coccyx |
| Proximal humerus | S42.2 | Fracture of upper end of humerus |
|  | S42.3 | Fracture of shaft of humerus |
|  | S42.4 | Fracture of lower end of humerus |
| Radius | S52.5 | Fracture of lower end of radius |
|  | S52.6 | Fracture of lower end of both ulna and radius |
| Ankle | S82.3 | Fracture of lower end of tibia |
|  | S82.5 | Fracture of medial malleolus |
|  | S82.6 | Fracture of lateral malleolus |
| Pelvis | S32.3 | Fracture of ilium |
|  | S32.4 | Fracture of acetabulum |
|  | S32.5 | Fracture of pubis |
|  | S32.7 | Multiple fractures of lumbar spine and pelvis |
|  | S32.8 | Fracture of other and unspecified parts of lumbar spine and pelvis |
| Rib | S22.3 | Fracture of rib |
|  | S22.4 | Multiple fracture of ribs |
|  | S22.5 | Flail chest |
| Distal femur | S72.4 | Fracture of lower end of femur |
| Clavicle | S42.0 | Fracture of clavicle |
| Hip | S72.0 | Fracture of head and neck of femur |
|  | S72.1 | Pertrochanteric fracture |

*ICD*, International Classification of Diseases; *NEC*, not elsewhere classified

**Supplementary Table 1** Comparison of demographics and baseline clinical characteristics between groups by time to treatment initiation (within 3 months and over 3 months groups)

|  | Initiation timing of osteoporosis medication | | |
| --- | --- | --- | --- |
|  | Within 3 months (n=4,421) | Over 3 months (n=1,464) | *p* value |
| **Time to treatment initiation (days)** |  |  |  |
| Mean±SD | 17.2±24.4 | 590.1±637.0 |  |
| Median (IQR) | 0 (0–33) | 335 (158–787) | <0.0001 |
| **Demographics** |  |  |  |
| **Patients, index year** |  |  | <0.0001 |
| 2003 | 132 (3.0) | 11 (0.8) |  |
| 2004 | 190 (4.3) | 46 (3.1) |  |
| 2005 | 250 (5.7) | 83 (5.7) |  |
| 2006 | 363 (8.2) | 96 (6.6) |  |
| 2007 | 372 (8.4) | 164 (11.2) |  |
| 2008 | 380 (8.6) | 145 (9.9) |  |
| 2009 | 432 (9.8) | 158 (10.8) |  |
| 2010 | 451 (10.2) | 137 (9.4) |  |
| 2011 | 444 (10.0) | 139 (9.5) |  |
| 2012 | 493 (11.2) | 163 (11.1) |  |
| 2013 | 483 (10.9) | 149 (10.2) |  |
| 2014 | 431 (9.8) | 173 (11.8) |  |
| **Sex** |  |  | 0.714 |
| Male | 653 (14.8) | 222 (15.2) |  |
| Female | 3,768 (85.2) | 1,242 (84.8) |  |
| **Age, years** |  |  |  |
| Mean±SD | 79.0±6.6 | 78.1±6.4 |  |
| Median (IQR) | 79 (74–84) | 78 (73–82) | <0.0001 |
| 60–64 | 27 (0.6) | 12 (0.8) | <0.0001 |
| 65–69 | 299 (6.8) | 108 (7.4) |  |
| 70–74 | 837 (18.9) | 338 (23.1) |  |
| 75–79 | 1,209 (27.3) | 406 (27.7) |  |
| 80–84 | 1,117 (25.3) | 376 (25.7) |  |
| 85–89 | 672 (15.2) | 161 (11.0) |  |
| 90+ | 260 (5.9) | 63 (4.3) |  |
| **Residence** |  |  | 0.059 |
| Metropolis | 1,984 (44.9) | 614 (41.9) |  |
| City | 791 (17.9) | 255 (17.4) |  |
| Rural | 1,646 (37.2) | 595 (40.6) |  |
| **Income** |  |  | <0.0001 |
| Medical aid (the lowest) | 546 (12.4) | 246 (16.8) |  |
| 1–4 | 1,063 (24.0) | 302 (20.6) |  |
| 5–8 | 1,205 (27.3) | 379 (25.9) |  |
| 9–10 (the highest) | 1,607 (36.4) | 537 (36.7) |  |
| **Type of insurance** |  |  | <0.0001 |
| Medical aid | 546 (12.4) | 246 (16.8) |  |
| Employee-insured | 2,641 (59.7) | 824 (56.3) |  |
| Self-employed, insured | 1,234 (27.9) | 394 (26.9) |  |
| **Disability** |  |  | 0.434 |
| None | 4,317 (97.6) | 1,436 (98.1) |  |
| Mild disability (grades 3–6) | 43 (1.0) | 9 (0.6) |  |
| Serious disability (grades 1–2) | 61 (1.4) | 19 (1.3) |  |
| **Medical utilization related to fracture or osteoporosis medication use** |  |  |  |
| Any osteoporotic fracture before first hip fracture (except for hip) | 1,410 (31.9) | 399 (27.3) | 0.001 |
| **Osteoporosis medication use before first hip fracture** |  |  |  |
| Ever used | 2,020 (45.7) | 595 (40.6) | 0.001 |
| Used within 1 year | 1,094 (24.7) | 310 (21.2) | 0.006 |
| **Comorbidities** |  |  |  |
| Diabetes mellitus | 1,200 (27.1) | 424 (29.0) | 0.177 |
| Hypertension | 2,694 (60.9) | 913 (62.4) | 0.331 |
| Dyslipidemia | 586 (13.3) | 235 (16.1) | 0.007 |
| Coronary heart disease | 510 (11.5) | 183 (12.5) | 0.321 |
| Cardiac dysrhythmia | 229 (5.2) | 73 (5.0) | 0.771 |
| PAOD | 364 (8.2) | 135 (9.2) | 0.240 |
| Kidney dysfunction | 82 (1.9) | 37 (2.5) | 0.113 |
| Parkinson’s disease | 130 (2.9) | 58 (4.0) | 0.054 |
| COPD | 572 (12.9) | 178 (12.2) | 0.440 |
| Rheumatoid arthritis | 226 (5.1) | 88 (6.0) | 0.185 |
| Cancer | 424 (9.6) | 162 (11.1) | 0.102 |
| Liver dysfunction | 312 (7.1) | 97 (6.6) | 0.574 |
| Stroke or TIA | 937 (21.2) | 352 (24.0) | 0.022 |
| Dementia | 641 (14.5) | 252 (17.2) | 0.012 |
| **CCI** |  |  | 0.001 |
| 0 | 1,095 (24.8) | 309 (21.1) |  |
| 1 | 1,215 (27.5) | 391 (26.7) |  |
| 2 | 852 (19.3) | 273 (18.7) |  |
| ≥3 | 1,259 (28.5) | 491 (33.5) |  |
| **Concomitant medications** |  |  |  |
| Acetaminophen | 1,404 (31.8) | 429 (29.3) | 0.079 |
| Antidepressants | 835 (18.9) | 285 (19.5) | 0.624 |
| Antiepileptics | 396 (9.0) | 164 (11.2) | 0.011 |
| Antipsychotics | 1,368 (30.9) | 396 (27.1) | 0.005 |
| Anxiolytics | 1,678 (38.0) | 573 (39.1) | 0.419 |
| CNS stimulants | 314 (7.1) | 119 (8.1) | 0.193 |
| COX-2 inhibitors | 1,207 (27.3) | 244 (16.7) | <0.0001 |
| HRT (women only) | 19 (0.4) | 7 (0.5) | 0.821 |
| Hypnotics | 608 (13.8) | 149 (10.2) | 0.0004 |
| Loop diuretics | 463 (10.5) | 138 (9.4) | 0.252 |
| MTX | 29 (0.7) | 13 (0.9) | 0.361 |
| NSAIDs | 3,854 (87.2) | 1,157 (79.0) | <0.0001 |
| Proton pump inhibitor | 566 (12.8) | 179 (12.2) | 0.566 |
| Steroids (including corticosteroids) | 764 (17.3) | 266 (18.2) | 0.438 |
| Thiazolidinediones | 48 (1.1) | 17 (1.2) | 0.811 |
| Thyroid therapy^a^ | 75 (1.7) | 30 (2.1) | 0.377 |

Data are presented as n (%) unless otherwise specified

*CCI*, Charlson Comorbidity Index; *CNS*, central nervous system; *COPD*, chronic obstructive pulmonary disease; *COX*, cyclooxygenase; *HRT*, hormone replacement therapy; *IQR*, interquartile range; *MTX*, methotrexate; *NSAID*, nonsteroidal anti-inflammatory drug; *PAOD*, peripheral arterial occlusive disease; *PPI*, proton pump inhibitor; *SD*, standard deviation; *TIA*, transient ischemic attack

^a^Includes levothyroxine sodium and a combination of levothyroxine and liothyronine sodium indicated for the treatment of hypothyroidism

**Supplementary Table 2** The effect of treatment duration with osteoporosis medication on subsequent osteoporotic fracture and mortality in the multiple treatment propensity score weighted model

| **Treatment duration** | SOF | Vertebral | Hip | Nonvertebral/nonhip | All-cause mortality | Death due to diseases of the respiratory system | Death due to diseases of the circulatory system | Death due to endocrine, nutritional, and metabolic diseases |
| --- | --- | --- | --- | --- | --- | --- | --- | --- |
| ST group | Reference | Reference | Reference | Reference | Reference | Reference | Reference | Reference |
| ED group | 0.94  (0.85-1.05) | 1.01  (0.87-1.17) | 0.94  (0.75-1.18) | 1.01  (0.86-1.19) | 0.90  (0.83-0.99) | 0.88  (0.67-1.15) | 0.91  (0.77-1.08) | 0.57  (0.39-0.84) |
| LD group | 1.01  (0.91-1.13) | 0.95  (0.82-1.11) | 1.17  (0.94-1.45) | 1.17  (1.00-1.36) | 0.70  (0.63-0.77) | 0.90  (0.68-1.17) | 0.58  (0.48-0.71) | 0.53  (0.35-0.78) |
| TC group | 0.84  (0.75-0.93) | 0.71  (0.60-0.84) | 0.68  (0.53-0.88) | 1.12  (0.95-1.32) | 0.63  (0.57-0.70) | 0.88  (0.66-1.16) | 0.33  (0.26-0.42) | 0.70  (0.48-1.01) |

Data are presented as propensity score-weighted hazard ratios with 95% confidence intervals

ST group, <3 months of treatment with osteoporosis medications; ED group, ≥3–<6 months of treatment with osteoporosis medications; LD group, ≥6–<12 months of treatment with osteoporosis medications; and TC group, ≥12 months of treatment with osteoporosis medications

**Supplementary Table 3 Sensitivity analysis for assessment of the risk of subsequent osteoporotic fracture among patients who have not experienced any osteoporotic fracture prior to initial hip fracture**

|  | SOF | | | Vertebral | | | Hip | | | Nonvertebral/nonhip | | |
| --- | --- | --- | --- | --- | --- | --- | --- | --- | --- | --- | --- | --- |
| **Treatment Duration** | Crude  HR | Adjusted HR* | Weighted HR | Crude  HR | Adjusted HR* | Weighted HR | Crude  HR | Adjusted HR* | Weighted HR | Crude  HR | Adjusted HR* | Weighted HR |
| **All** |  |  |  |  |  |  |  |  |  |  |  |  |
| ST group | Reference | Reference | Reference | Reference | Reference | Reference | Reference | Reference | Reference | Reference | Reference | Reference |
| ED group | 1.08  (0.86-1.36) | 1.05  (0.83-1.33) | 1.05  (0.92-1.19) | 1.19  (0.86-1.66) | 1.20  (0.85-1.68) | 1.18  (0.98-1.42) | 0.94  (0.58-1.54) | 0.87  (0.52-1.43) | 0.94  (0.71-1.24) | 1.25  (0.89-1.76) | 1.19  (0.84-1.69) | 1.19  (0.98-1.44) |
| LD group | 1.07  (0.85-1.35) | 0.95  (0.74-1.22) | 0.99  (0.86-1.13) | 0.93  (0.66-1.31) | 0.80  (0.55-1.17) | 0.83  (0.67-1.01) | 1.12  (0.70-1.77) | 0.95  (0.58-1.57) | 1.14  (0.88-1.49) | 1.32  (0.94-1.84) | 1.13  (0.78-1.63) | 1.15  (0.95-1.40) |
| TC group | 0.92  (0.67-1.26) | 0.81  (0.57-1.15) | 0.82  (0.71-0.94) | 1.01  (0.65-1.59) | 0.83  (0.50-1.35) | 0.83  (0.68-1.02) | 1.03  (0.55-1.93) | 0.92  (0.46-1.83) | 0.90  (0.67-1.20) | 0.87  (0.53-1.45) | 0.78  (0.45-1.34) | 0.79  (0.63-0.98) |
| **Male** |  |  |  |  |  |  |  |  |  |  |  |  |
| ST group | Reference | Reference | Reference | Reference | Reference | Reference | Reference | Reference | Reference | Reference | Reference | Reference |
| ED group | 1.02  (0.53-1.96) | 1.24  (0.58-2.65) | 1.07  (0.74-1.53) | 1.92  (0.76-4.87) | 6.76  (1.04-43.88) | 2.00  (1.18-3.37) | 0.94  (0.30-3.01) | 1.55  (0.17-14.06) | 1.01  (0.53-1.92) | 0.77  (0.25-2.39) | 0.76  (0.20-2.93) | 0.73  (0.39-1.39) |
| LD group | 0.54  (0.23-1.31) | 0.68  (0.23-2.03) | 0.61  (0.39-0.96) | 0.27  (0.04-2.12) | 0.08  (0.00-4.60) | 0.35  (0.13-0.90) | 0.86  (0.24-3.12) | 0.06  (0.00-8.00) | 0.92  (0.46-1.85) | 0.71  (0.20-2.50) | 0.92  (0.17-5.05) | 0.67  (0.34-1.35) |
| TC group | 1.22  (0.47-3.15) | 1.16  (0.38-3.59) | 1.20  (0.81-1.77) | 1.37  (0.30-6.25) | 8.31  (0.48-143.54) | 1.01  (0.51-2.01) | 0.76  (0.10-5.90) | 0.16  (0.01-4.12) | 0.56  (0.23-1.37) | 1.26  (0.28-5.62) | 1.00  (0.14-7.27) | 1.69  (0.96-2.98) |
| **Female** |  |  |  |  |  |  |  |  |  |  |  |  |
| ST group | Reference | Reference | Reference | Reference | Reference | Reference | Reference | Reference | Reference | Reference | Reference | Reference |
| ED group | 1.07  (0.84-1.38) | 1.03  (0.80-1.34) | 1.03  (0.89-1.18) | 1.08  (0.76-1.53) | 1.05  (0.73-1.51) | 1.07  (0.87-1.30) | 0.97  (0.57-1.68) | 0.95  (0.54-1.67) | 0.95  (0.69-1.29) | 1.28  (0.89-1.85) | 1.23  (0.85-1.80) | 1.21  (0.98-1.49) |
| LD group | 1.10  (0.86-1.40) | 0.97  (0.75-1.27) | 1.00  (0.87-1.16) | 0.92  (0.64-1.31) | 0.76  (0.51-1.13) | 0.82  (0.66-1.01) | 1.19  (0.72-1.98) | 1.08  (0.62-1.87) | 1.21  (0.90-1.62) | 1.34  (0.94-1.92) | 1.18  (0.80-1.74) | 1.17  (0.95-1.44) |
| TC group | 0.87  (0.62-1.23) | 0.77  (0.53-1.11) | 0.76  (0.65-0.89) | 0.93  (0.58-1.49) | 0.72  (0.42-1.21) | 0.77  (0.62-0.96) | 1.11  (0.57-2.17) | 1.11  (0.53-2.31) | 0.97  (0.71-1.33) | 0.82  (0.48-1.41) | 0.73  (0.41-1.30) | 0.69  (0.54-0.88) |

Data are presented as hazard ratios with 95% confidence intervals

ST group, <3 months of treatment with osteoporosis medications; ED group, ≥3–<6 months of treatment with osteoporosis medications; LD group, ≥6–<12 months of treatment with osteoporosis medications; and TC group, ≥12 months of treatment with osteoporosis medications

*Adjusted for the index year, sex, age group, residence, income, type of insurance, disability, any osteoporotic fracture before the initial hip fracture, use of osteoporosis medications before the initial hip fracture, comorbidities, and concurrent medications listed in the baseline table

**Supplementary Table 4 Sensitivity analysis for assessment of the risk of death among patients who have not experienced any osteoporotic fracture prior to initial hip fracture**

|  | All-cause mortality | | | Death due to diseases of the respiratory system | | | Death due to diseases of the circulatory system | | | Death due to endocrine, nutritional, and metabolic diseases | | |
| --- | --- | --- | --- | --- | --- | --- | --- | --- | --- | --- | --- | --- |
| **Treatment Duration** | Crude  HR | Adjusted HR* | Weighted HR | Crude  HR | Adjusted HR* | Weighted HR | Crude  HR | Adjusted HR* | Weighted HR | Crude  HR | Adjusted HR* | Weighted HR |
| **All** |  |  |  |  |  |  |  |  |  |  |  |  |
| ST group | Reference | Reference | Reference | Reference | Reference | Reference | Reference | Reference | Reference | Reference | Reference | Reference |
| ED group | 0.86  (0.71-1.04) | 0.87  (0.71-1.05) | 0.86  (0.77-0.96) | 0.98  (0.57-1.66) | 0.98  (0.56-1.72) | 0.87  (0.64-1.20) | 0.82  (0.57-1.17) | 0.81  (0.56-1.17) | 0.91  (0.74-1.12) | 0.49  (0.19-1.25) | 0.36  (0.13-1.03) | 0.42  (0.25-0.72) |
| LD group | 0.58  (0.47-0.72) | 0.64  (0.51-0.81) | 0.65  (0.58-0.73) | 0.82  (0.47-1.42) | 0.92  (0.50-1.71) | 0.90  (0.65-1.23) | 0.41  (0.26-0.64) | 0.47  (0.29-0.76) | 0.48  (0.38-0.62) | 0.55  (0.23-1.34) | 0.37  (0.14-1.02) | 0.46  (0.27-0.77) |
| TC group | 0.54  (0.39-0.73) | 0.61  (0.44-0.84) | 0.55  (0.48-0.63) | 0.39  (0.14-1.09) | 0.37  (0.12-1.13) | 0.44  (0.30-0.66) | 0.39  (0.20-0.75) | 0.46  (0.23-0.93) | 0.44  (0.34-0.58) | 0.37  (0.09-1.61) | 0.29  (0.06-1.47) | 0.29  (0.16-0.55) |
| **Male** |  |  |  |  |  |  |  |  |  |  |  |  |
| ST group | Reference | Reference | Reference | Reference | Reference | Reference | Reference | Reference | Reference | Reference | Reference | Reference |
| ED group | 1.31  (0.87-1.96) | 1.36  (0.85-2.18) | 1.22  (0.96-1.54) | 1.69  (0.68-4.20) | 2.02  (0.55-7.38) | 1.56  (0.91-2.68) | 1.05  (0.36-3.01) | 1.86  (0.22-15.60) | 1.24  (0.68-2.25) | NA | NA | NA |
| LD group | 1.35  (0.88-2.08) | 1.10  (0.64-1.91) | 1.31  (1.03-1.66) | 1.74  (0.68-4.50) | 1.27  (0.24-6.65) | 1.74  (1.01-3.00) | 0.51  (0.11-2.28) | 0.46  (0.04-5.81) | 0.42  (0.17-1.04) | 1.41  (0.26-7.70) | NA | 1.22  (0.42-3.56) |
| TC group | 1.09  (0.56-2.13) | 1.07  (0.48-2.41) | 1.03  (0.78-1.36) | 0.63  (0.08-4.89) | 2.40  (0.16-36.42) | 1.21  (0.64-2.30) | 1.24  (0.28-5.62) | 0.92  (0.03-27.51) | 0.79  (0.37-1.69) | NA | NA | NA |
| **Female** |  |  |  |  |  |  |  |  |  |  |  |  |
| ST group | Reference | Reference | Reference | Reference | Reference | Reference | Reference | Reference | Reference | Reference | Reference | Reference |
| ED group | 0.80  (0.65-0.99) | 0.81  (0.65-1.01) | 0.81  (0.72-0.92) | 0.87  (0.45-1.67) | 0.85  (0.41-1.73) | 0.75  (0.51-1.11) | 0.77  (0.53-1.12) | 0.73  (0.49-1.09) | 0.85  (0.68-1.06) | 0.59  (0.22-1.54) | 0.42  (0.14-1.30) | 0.48  (0.28-0.83) |
| LD group | 0.49  (0.39-0.63) | 0.57  (0.44-0.74) | 0.56  (0.49-0.64) | 0.70  (0.35-1.39) | 0.81  (0.37-1.75) | 0.77  (0.52-1.13) | 0.39  (0.24-0.62) | 0.44  (0.26-0.73) | 0.47  (0.36-0.61) | 0.46  (0.16-1.28) | 0.25  (0.07-0.86) | 0.37  (0.20-0.67) |
| TC group | 0.48  (0.34-0.67) | 0.56  (0.39-0.81) | 0.50  (0.43-0.58) | 0.38  (0.11-1.27) | 0.33  (0.09-1.24) | 0.32  (0.19-0.54) | 0.31  (0.15-0.65) | 0.38  (0.18-0.83) | 0.39  (0.30-0.53) | 0.43  (0.10-1.89) | 0.35  (0.06-1.95) | 0.32  (0.17-0.61) |

Data are presented as hazard ratios with 95% confidence intervals

ST group, <3 months of treatment with osteoporosis medications; ED group, ≥3–<6 months of treatment with osteoporosis medications; LD group, ≥6–<12 months of treatment with osteoporosis medications; and TC group, ≥12 months of treatment with osteoporosis medications

*Adjusted for the index year, sex, age group, residence, income, type of insurance, disability, any osteoporotic fracture before the initial hip fracture, use of osteoporosis medications before the initial hip fracture, comorbidities, and concurrent medications listed in the baseline table

**Supplementary Table 5 Risk of subsequent osteoporotic fracture and mortality on treatment duration as continuous supply days**

| **Treatment Duration** | SOF | | | All-cause mortality | | |
| --- | --- | --- | --- | --- | --- | --- |
|  | Crude HR | Adjusted HR* | Weighted HR | Crude HR | Adjusted HR* | Weighted HR |
| All | 0.99 (0.82-1.21) | 0.80 (0.65-1.00) | 0.87 (0.79-0.96) | 0.51 (0.42-0.62) | 0.61 (0.49-0.75) | 0.61 (0.55-0.67) |
| Male | 0.82 (0.42-1.63) | 0.99 (0.44-2.24) | 0.86 (0.62-1.18) | 1.05 (0.69-1.59) | 0.97 (0.57-1.65) | 0.99 (0.80-1.22) |
| Female | 0.98 (0.80-1.21) | 0.79 (0.63-1.00) | 0.86 (0.78-0.95) | 0.45 (0.37-0.56) | 0.55 (0.43-0.70) | 0.54 (0.49-0.60) |

*Adjusted for the index year, sex, age group, residence, income, type of insurance, disability, any osteoporotic fracture before the initial hip fracture, use of osteoporosis medications before the initial hip fracture, comorbidities, and concurrent medications listed in the baseline table

**Supplementary Figure 1. Distribution of absolute standardized mean difference for multinominal propensity score**


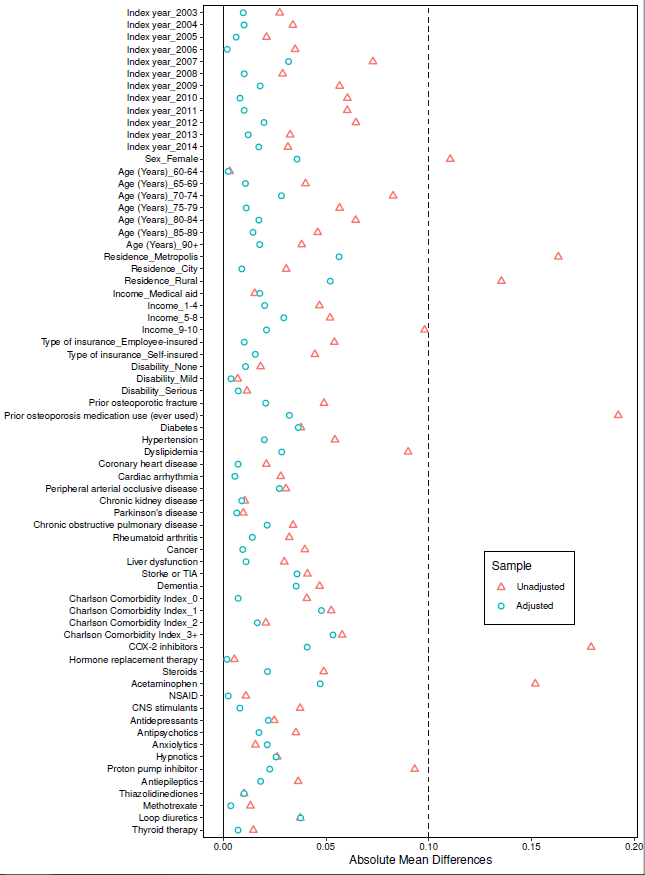

Supplement: Supplementary file 1 — Supplementary file1 (DOCX 158 KB) [file 11657_2024_1366_MOESM1_ESM.docx]
